# tRNA-derived small RNAs in disease immunity

**DOI:** 10.7150/thno.102650

**Published:** 2025-01-01

**Authors:** Hongyuan Jia, Linling Zhang

**Affiliations:** 1 Department of Radiation Oncology, Sichuan Clinical Research Center for Cancer, Sichuan Cancer Hospital & Institute, Sichuan Cancer Center, Affiliated Cancer Hospital of University of Electronic Science and Technology of China, Chengdu, China.; 2 Department of Respiratory and Critical Care, Chengdu Third People's Hospital, Chengdu, China.

**Keywords:** tRNA-derived small RNA, non-coding RNA, biogenesis, adaptive immunity, tumor immunology, autoimmune diseases

## Abstract

Recently, members of a unique species of non-coding RNA, known as transfer RNA-derived small RNAs (tsRNAs) have been reported to serve multiple molecular functions, including in cells that mediate immunity. Because of their low molecular weights, tsRNAs were previously difficult to detect and were thus overlooked, until now. In this review, we delve into the biogenesis of tsRNAs and their diverse biological functions, ranging from transcriptional regulation to modulation of mRNA translation. We highlight the current evidence demonstrating their involvement in the immune response, as well as how tsRNAs modulate immunity to influence tumor growth and spread, autoimmune disease pathology and infection by pathogens. We surmise that tsRNAs are likely informative as diagnostic markers of cellular homeostasis and disease, and that therapeutic targeting of tsRNAs could be beneficial for a range of human diseases. Improved knowledge on the functions for tsRNAs in the mammalian immune system will enable us to leverage tsRNAs for their effective clinical use as treatments for human health challenges.

## Introduction

Transfer RNAs (tRNAs) are indispensable molecular components that comprise the cellular protein synthesis machinery of cells, serving to direct the accurate assembly of polypeptides 1, 2. Recently, the traditional view of tRNAs as mere translators of genetic information has been challenged by the discovery of tRNA-derived small RNAs (tsRNAs) which, remarkably, have revealed further regulatory complexity for tRNAs and their functional products within cells. Indeed, tsRNAs, generated through alternative processing pathways, have been found to influence gene expression and mediate cellular responses to stress 3-7. Studies on how tsRNAs exert their molecular functions in cells of the immune system has begun to reveal how they influence signaling pathways. For example, tsRNAs have been shown to play a role in the activation of immune cells and the production of cytokines, which are crucial for mounting an effective immune response against a pathogen challenge 8, 9. Moreover, tsRNAs have also been postulated to function as modulators of the immune responses in cancer, particularly in tumor growth and immune evasion 10. Furthermore, tsRNAs have also been suggested to play a critical role in autoimmune disorders 11. Here, we will review our current understanding of the role of tsRNAs in cells of the immune system and how they are crucial to health and disease.

## Biogenesis and classification of tsRNA

**Figure 1:**
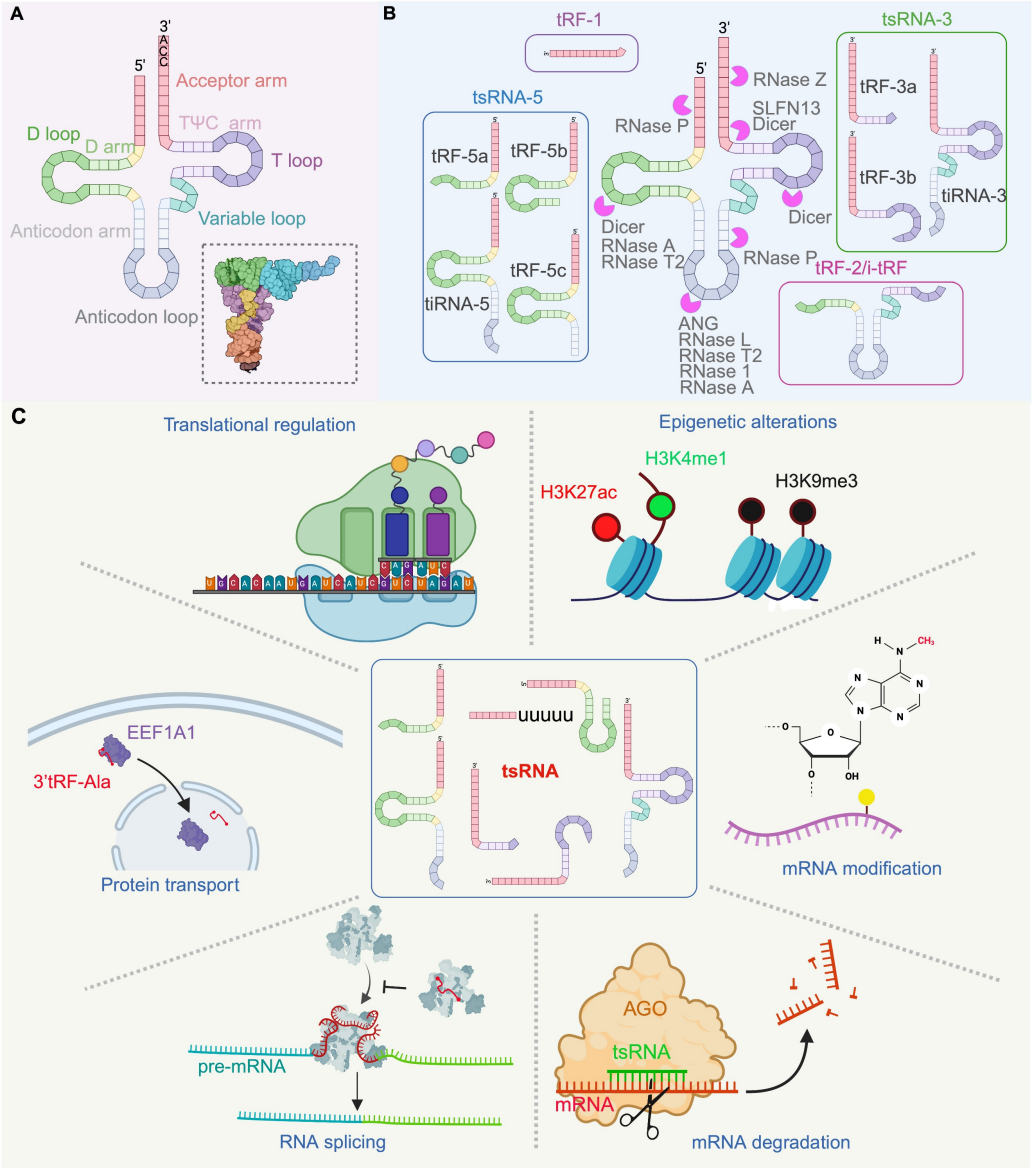
**Structure, classification and function of tRNA-derived small RNA (tsRNA). (A)** tRNA structure: The tRNA mocular consists of four arms (acceptor arm, D arm, TΨC arm and anticodon arm), three loops (D loop, TΨC loop and anticodon loop) and a variable loop structure. The L-shaped tertiary structure model of tRNA is depicted in the lower right corner. **(B)** Classification and Biogenesisof tsRNA: tsRNA is primarily generated by specific nucleases (such as ANG, Dicer, SLFN13, RNase 1, RNase P, RNase Z, RNase T2, RNaseA, RNase L, etc.) that cleave mature tRNAs or during the processing of tRNA precursors. **(C)** Function of tsRNA: tsRNA participates in the regulation of numerous of biological processes, including epigenetic alterations, mRNA modifications, gene sciencing, RNA splicing, translational regulation and protein transport. For example, the 3'tRF-Val directly binds to the chaperone molecule EEF1A1, an interaction that does not affect the levels of EEF1A1 mRNA and protein but mediates its transport to the nucleus. This figure was created with BioRender.com.

The biogenesis of tsRNA involves the transcription of tRNA genes by RNA polymerase III, followed by the subsequent maturation of pre-tRNAs into mature tRNAs with a “cloverleaf” structure which, in turn, folds further into an L-shaped conformation (Figure 1A) 1, 12-15. This molecular assembly process includes the removal of 5' and 3' sequences by RNase P and RNase Z or ELAC2, respectively, as well as the addition of a 5'-CCA-3' tail by a nucleotidyl transferase. Mature tRNAs fold into an L-shaped conformation with exposed regions that are susceptible to cleavage, often at the anticodon or loop regions, by various enzymes including RNase P, Dicer, and ANG 3, 16-22. Also, tRNA cleavage is known to occur outside of the loop regions. Other enzymes that can cleave tRNA to generate tsRNA include RNase 1, RNase Z, and members of the RNase A family like ANG 23-25, RNase T2 26, 27, and certain interferon-stimulated genes, such as RNase L, Schlafen 11 (SLFN11) and Schlafen 13 (SLFN13) 28-30. However, the exact cleavage mechanisms and sites for some of these enzymes, remain under investigation. Notably, the presence of tsRNAs appears to be closely linked to chemical modifications of tRNAs, with over 170 distinct modifications identified 31-36. Such modifications, including cytosine-C5 methylation by DNMT2 and NSUN2, enhance the stability of tRNAs as well as translational decoding 37, 38. Also, it has been found that tRNAs that are deficient in chemical modifications are more likely to be cleaved into tsRNA. For instance, the m5C38 modification of target tRNAs by DNMT2 can reduce the generation of 5' tRNA halves under stress 39, 40. Furthermore, reduced levels of m5C48/49N modification arising from a deficiency in the modifier known as SUN2 can lead to increased levels of 5' tRNA halves 41. The absence of certain modification-related enzymes such as PUS7, TRMT10A, ALKBH3, and BCDIN3D can also alter the relative composition of tsRNA species and their abundance within cells 22, 42-45. Collectively, these observations underscore the impact of tRNA modifications on tsRNA biogenesis and in the event that there is no significant change to the quantity of parental tRNA species from which these are derived. What remains unclear is the entire lexicon of sequence motifs recognised by RNA-bodifying enzymes, the full complexity of modification states of tsRNAs, as well as how targeting enzymes facilitate tsRNA biogenesis. Moreover, whether modification-dependent protein interactions or tRNA folding dynamics regulate the susceptibility of tRNA to ribonuclease activity, all also remain to be clarified.

Currently, tsRNAs can be classified along two main groups based on size and origin: tRNA halves (tiRNAs) and tRNA-derived fragments (tRFs) (Figure 1B). For tiRNAs, these arise from the cleavage at the anticodon loop of mature tRNAs and are further divided into 5' tiRNAs (30-35 nt) and 3' tiRNAs (40-50 nt) 46, 47. In contrast, tRFs are shorter in length, ranging from 13 to 30 nt, and include specific types such as 3U-tRF (tRF-1), tRF-3, tRF-5, and internal tRF (i-tRF or tRF-2), while tRF-1 originates from the 3' end of pre-tRNAs and varies in length. Of note, tRF-3, derived from the TΨC loop of mature tRNAs, is divided into tRF-3a (~18 nt) and tRF-3b (~22 nt), while tRF-5 extends from the tRNA terminus, and is subclassified further as tRF-5a (14-16 nt), tRF-5b (22-24 nt), and tRF-5c (27-30 nt). The i-tRFs are derived from anticodon loops of mature tRNAs.

## Functional mechanisms of tsRNAs

### Transcriptional regulation

Increasingly, tsRNAs have emerged as significant modulators of transcription through epigenetic mechanisms, that is, through gene expression changes without DNA sequence alterations 48, 49. Indeed, tsRNAs have been described in influence DNA methylation, histone modification, and non-coding RNA regulation, all of which control gene expression at the transcriptional level to mediate cellular function in health and disease 50. In the context of non-small cell lung cancer (NSCLC), it has been reported that upregulation of tsRNA AS-tDR-007333 in NSCLC cells could promote cell proliferation and migration by influencing histone modifications at the MED29 promoter 51. In another example, the tsRNA known as tRF-GG has been found to influence the production of non-coding RNAs, a function that is influenced by the stability and activity of Cajal bodies within cells. Indeed, tRF-GG was reported to modulate the transcriptional repression of MERVL elements by heterochromatin through U7 snRNA regulation which, in turn, impacted histone protein availability and led to gene regulation effects 52. Furthermore, tsRNAs have been found to interact with PIWI proteins, suggesting they could be relevant to gene silencing. For example, a tRF-5c species derived from tRNA-Glu has been reported to interact with Piwi-like protein 4, so as to recruit chromatin-modifying enzymes to the CD1A promoter which, in turn, enhances H3K9 methylation, so as to suppress CD1A gene transcription 53. Other tsRNAs have also been identified in complexes with Piwi-like protein 2, suggestive of their influence kin downstream gene transcription 54. Further research is needed to understand these mechanisms fully.

### Post-transcriptional regulation

The roles for tsRNAs in post-transcriptional regulatory functions can, in part, be attributable to their document interaction with Argonaute (AGO) proteins 55. Indeed, tsRNAs are capable of forming RNA-induced silencing complexes (RISC) with AGO proteins to regulate target RNA expression through base-pairing, a process that is thought to involve Dicer 56-59. For example, tRF3008A from tRNA-Val interacts with AGO proteins to destabilize the FOXK1 transcript in colorectal cancer (CRC) cells, and this leads to reduced proliferation 60. Notably, tsRNAs interact with specific AGO proteins, with some studies showing tsRNAs binding to AGO1, 3, and 4 61; with others studies showing interactions with AGO2 58, 62. Furthermore, tsRNAs are involved in the regulation of m6A methylation, an epigenetic modification that modulates target gene expression. For example, tRF-22 interacts with the 3' UTR of METTL3 mRNA in an AGO2-dependent manner, so as to modulate m6A methylation activity which, in turn, affects the expression of Axin1 and Arid1b in cells 63.

Beyond their roles in RISC complex assembly and function, tsRNAs also interact with RNA-binding proteins (RBPs) to sequester them from interacting with other RNAs, leading to changes in the landscape for mRNA stability within cells 64-67. Indeed, as an example, i-tRFs from multiple tRNAs can interact with YBX1, leading to the destabilization of oncogenic transcripts in breast cancer (BC) cells 64. Similarly, tRF-5 from tRNA-Gln interacts with IGF2BP1 to displace IGF2BP2-bound c-MYC RNA, leading to reduced mRNA stability of c-MYC transcripts 68, 69. Moreover, tsRNAs also regulate protein transport, such as in the case of 3'tRF-Val which has been shown to bind EEF1A1 and mediate the nuclear transport of RBPs and modulating p53 signaling pathways 70. In another such example, tRF-29-79 facilitates the cytoplasmic transport of the RNA-binding protein PTBP1 which, in turn, affects alternative splicing of SLC1A5 71. In addition to protein transport, tsRNAs have also been found to modulate target protein stability, such as in the case of tiRNA-Gly that interacts with RBM17 so as to enhance its prevalence within cells through inhibiton of proteasomal degradation 72, as well as in the case of tiRNA-Val-CAC-2 which stabilizes FUBP1 protein within cells while concomitantly promoting c-MYC transcription 73.

### Translational regulation

Current evidence indicates that tsRNAs play a significant role in the translational regulation of protein synthesis, a critical process that includes transcription, mRNA processing, and ribosomal translation 74, 75. Indeed, various reports have indicated that tsRNAs modulate the translation of proteins 24, 76-78, such as when under stress conditions like hypoxia or nutrient deprivation, where cells repress translation to conserve on intracellular resources. In that scenario, tsRNAs such as 5'tiRNAs, have been shown to interact with proteins like YBX1, a member of the cold shock family of proteins and an essential regulator of transcription and translation 79, which can inhibit global protein translation by promoting stress granule formation 80. Interestingly, such tiRNAs contain a TOG motif that forms G-quadruplex structures, important for translational repression 81, 82. Expanding on this notion, Lyons *et al.* discovered that G4-tiRNAs target the HEAT1 domain of eIF4G, leading to impaired ribosome scanning on mRNAs and the formation of stress granules 83. Moreover, tRF-5 can inhibit translation by sequestering PABPC1, in the context of PUS7-dependent ψ8 modification 42, while a tRF-1, tRF-Gln-CTG-026, can repress global protein synthesis by affecting the association between TSR1 and the pre-40S ribosome 76. In addition to nuclear genome-derived tsRNA species, mitochondrial tsRNAs have also been reported to participate in translational regulation, such as in the case of 5'tsRNA-Glu-CTC, a mitochondrial tsRNA that disrupts mt-tRNA-Leu aminoacylation as well as the translation of mitochondria-encoded proteins 84. In contrast to the tsRNAs that inhibit translation, some tsRNAs, such as 3'tsRNA-Leu-CAG, can enigmatically enhance translation and promote pre-18S ribosomal RNA processing, as well as increase the availability of 40S ribosomal subunits within cells 77, 85. These lines of evidence speak to the versatile roles of tsRNAs in controlling protein synthesis in homeostasis and stress.

A summary illustration for how tsRNAs contribute to gene expression, is provided in Figure 1C.

## The role of tsRNAs in immunity

The immune system of mammals encompassing a variety of immune cells and organs, altogether representing an intricate defense mechanism that protects against infection and illness 86, 87. Comprising both innate and adaptive immunity, it is responsible for the identification and elimination of foreign pathogens such as bacteria, viruses, and parasites, while also monitoring and clearing abnormal cells within the body 86, 87. Recent studies have revealed the presence of tsRNAs within immune cells 88, including candidate tsRNAs that influence myeloid cell differentiation. For example, the tsRNA known as 5'tiRNA-Pro-CGG-1, found in extracellular vesicles from osteoblasts, has been shown to enhance protein translation, cell proliferation, and myeloid differentiation when transferred to granulocyte-monocyte progenitors 89. This suggests a potential role for tsRNA in immune modulation.

### Functions for tsRNAs in innate immunity

**Figure 2:**
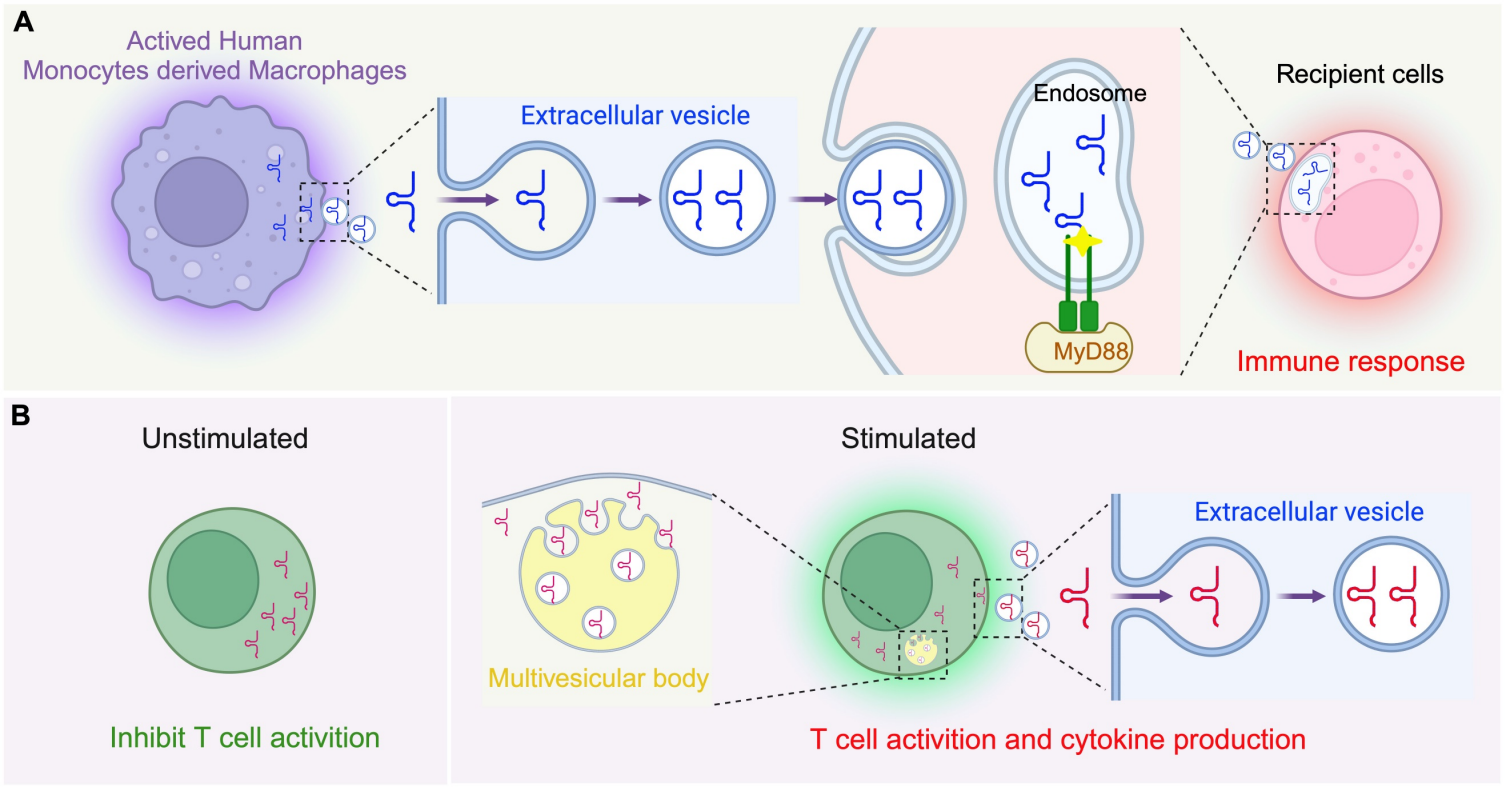
**Regulation of immune cell function by tsRNA. (A)** In innate immunity, activated human monocyte-derived macrophages secrete extracellular vesicles (EVs) containing tsRNA, particularly EV-5'-tRNA-His-GUG half molecules, to activate the immune response. The 5'-tRNA-Val-CAC/AAC half-molecule effectively targets and eliminates intracellular pathogens by activating TLR7. **(B)** In adaptive immunity, T cell activation triggers the formation of multivesicular body (MVB) and the release of a specific set of tRFs via EVs derived from the 5' end and 3' inner region of trRNA without variable loops. This figure was created with BioRender.com.

The innate immune system acts as the frontline defense of the body against invading pathogens, utilizing both physical barriers like the skin and mucous membranes, and cellular components such as leukocytes to initiate a swift defense 90, 91. A critical element of this system is the Toll-like receptor (TLR) family, with TLR7 being particularly significant for its ability to detect single-stranded RNAs from foreign pathogens. Our understanding of the specific endogenous RNAs that activate TLR7 has remained poor until recent findings, as follows. Pawar *et al.* identified that 5'-tRNA half molecules are key activators of TLR7 92, such that, during mycobacterial infections, TLR7 activation on the cell surface leads to an upregulation of 5'-tRNA half molecules in human monocyte-derived macrophages (HMDMs), leading to their accumulation within extracellular vesicles (EVs), with specific tsRNA species such as EV-5'-tRNA-His-GUG showing higher concentrations than the most prevalent EV-microRNAs (Figure 2A) 92. Notably, the mechanism by which these molecules are transferred to recipient cells so as to activate TLR7 within endosomes has been experimentally confirmed as a new pathway for tRNA in mediating innate immunity, and 5'-tRNA half molecules are now recognizes as “immune activators”. In another example, the 5'-tRNA-Val-CAC/AAC half, prevalent in macrophage EVs, has been demonstrated to robustly activate TLR7 as part of a molecular mechanism that mediates bacterial clearance, and this function relies on the terminal GUUU sequence of this tsRNA 93. Thus, these findings have expanded our understanding of tsRNA as intrinsic ligands for TLR7 and their active participation in signalling the innate immune response.

### Functions for tsRNAs in adaptive immunity

Adaptive immunity provides specific responses through B and T cells, offering long-term protection and forming immunological memory to rapidly respond to the same pathogens upon re-exposure 94, 95. Recent studies have suggested that tsRNAs have the potential to modulate T cell activation. Chiou *et al.* discovered a significant enrichment of tsRFs in EVs released by T cells, with 45% of tsRNAs being more abundant than miRNAs 96. Upon activation, T cells induced the release of a distinct subset of tsRNAs derived from specific regions within their parental tRNAs, excluding variable loops, via EVs. Disruption of EV biogenesis pathways results in the intracellular retention of these tRFs within multivesicular bodies (MVBs). The use of antisense oligonucleotides to target these tsRNAs has been shown to enhance T cell activation, indicating a selective release mechanism of tRFs into EVs via MVBs, potentially removing suppressive factors within the immune response (Figure 2B) 96. While these insights have broadened our understanding of tsRNAs in the adaptive immune response, how tsRNAs mediate the complex dynamics of immune cell functions remains to be better clarified.

## The roles for tsRNAs in mediating immunity in disease

### The roles for tsRNAs in tumor immunology

**Figure 3:**
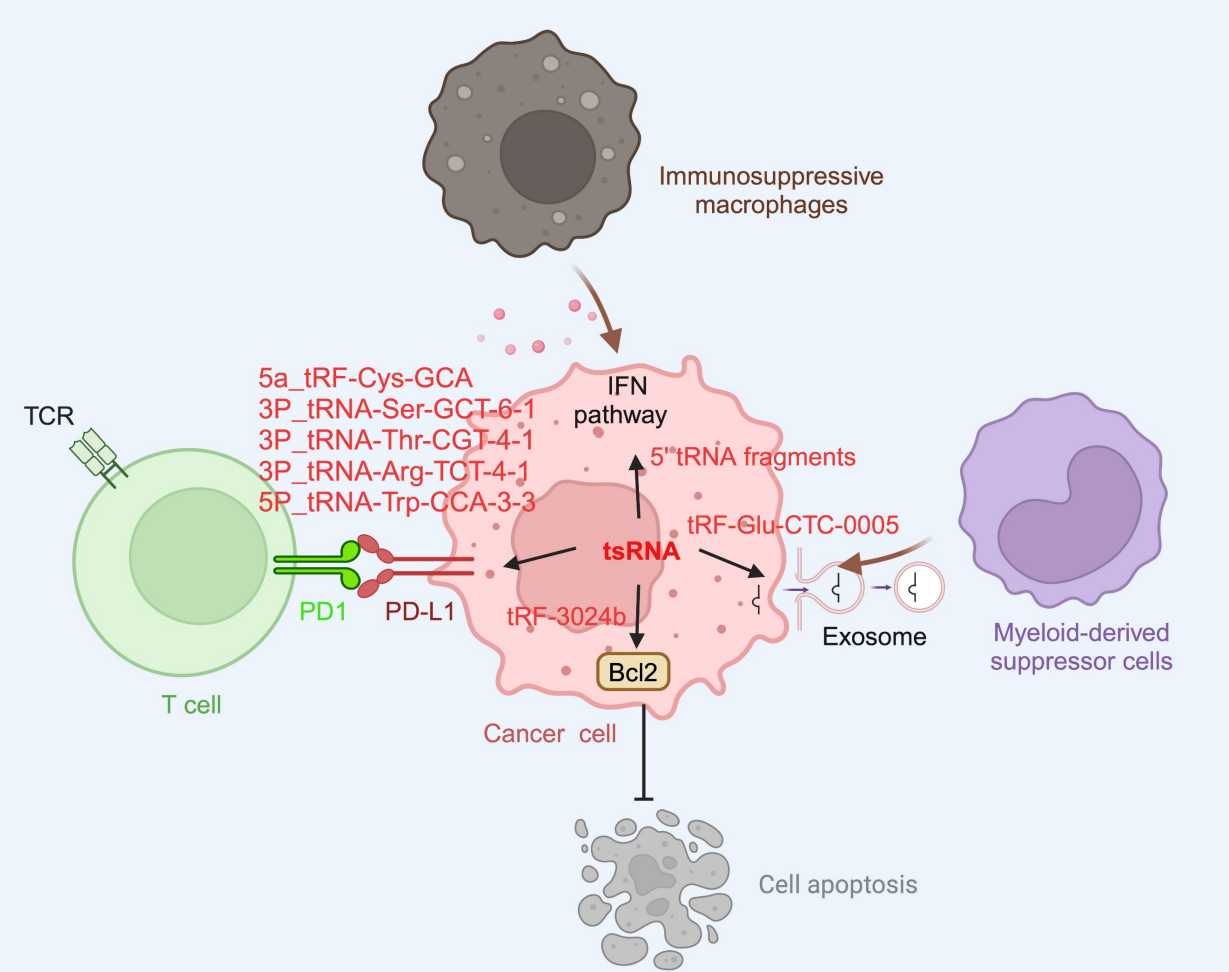
** tsRNA in tumor immunology.** tsRNA within tumor cells contribute to immune evasion by enhancing the expression of PD-L1. Specific tsRNA species, including 5a_tRF-Cys-GCA, 3P_tRNA-Ser-GCT-6-1, 3P_tRNA-Thr-CGT-4-1, 3P_tRNA-Arg-TCT-4-1, and 5P_tRNA-Trp-CCA-3-3, have been identified to play a role in this process. However, the precise molecular mechanisms remain unclear. Additionally, tsRNA can recruit immunosuppressive macrophagesand myeloid-derived suppressor cells by modulating the interferon pathway, as exemplified by 5'-tRNA fragments. They also contribute to the formation of an immunosuppressive microenvironment by secreting exosomes containing tsRNAs, such as tRF-Glu-CTC-0005. Furthermore, tsRNAs can impede the effectiveness of immunotherapy by inhibiting apoptosis. For instance, tRF-3024b reduces tumor cell apoptosis by promoting BCL-2 expression, thereby inhibiting the therapeutic effects of immune checkpoint inhibitors. This figure was created with BioRender.com.

Cancer is a leading cause of mortality and a significant factor in reducing life expectancy globally. In 2020, it was estimated that there were 19.3 million new cancer cases worldwide, excluding non-melanoma skin cancer, and nearly 10.0 million cancer-related deaths 97. Understanding the pathogenesis of cancer and developing better diagnostics and effective treatment strategies for this condition is an urgent priority worldwide. It has been recognized that the avoidance of immune destruction is one of the 14 hallmarks of cancer, and this underscores the indispensable role of the immune system in cancer development and progression 98. Recent studies have shed light on the regulatory role of tsRNAs in tumor proliferation 70, 99, 100, apoptosis 66, 70, and tumor metastasis 72, 101-103, all features of which influence cancer development and severity. Moreover, the association of tsRNAs with tumor immunity has also been reported (Figure 3). For instance, a study by Shan *et al.* demonstrated a significant link between T cell activation and the abundance of tsRNAs such as ts-34 and ts-49, in breast cancer patients 104. In that study, it was reported subjects with T cell exhaustion had low levels of ts-34 or high levels of ts-49 which were associated with enhanced survival rates 104. In lung cancer, tsRNAs such as 5a_tRF-Cys-GCA, 3P_tRNA-Ser-GCT-6-1, 3P_tRNA-Thr-CGT-4-1, 3P_tRNA-Arg-TCT-4-1, and 5P_tRNA-Trp-CCA-3-3 were correlated with the PD-L1 immune checkpoint and PD-L1 signaling pathway-related genes 105. These findings indicate that the presence of tsRNAs are relevant to cancer and, arguably, targeting such tsRNAs might have potential therapeutic significance when treating this condition.

Recent studies have shed light into the mechanisms through which tsRNA regulates tumor immunity. In prostate cancer, METTL1 depletion results in the emergence of a novel class of 5' tsRNAs, which modulate translation control to favor the synthesis of key regulators of tumor growth suppression, interferon pathways, and immune effectors 106. In esophageal squamous cell carcinoma (ESCC), tRF-3024b, which upregultes in ESCC cells that survived co-culture with cytotoxic T lymphocytes (CTLs), has been shown to reduce tumor cell apoptosis by sequestering miR-192-5p and promoting BCL-2 expression, thereby enhancing the protective effects of BCL-2 107. This finding is suggestive of an approach through which modulation of key tsRNAs could enhance the response of ESCC to CTLs as an strategy to improve the effectiveness of immunotherapy for ESCC. In another example, 3'tRF-Ala-AGC is upregulated in BC specimens as well as in adriamycin-resistant cancer cells, and this tsRNA has been linked to the promotion of malignant cell activity and the facilitation of M2 macrophage polarization via interacting with the Type 1-associated death domain protein (TRADD). Moreover, it was found that overexpression of 3'tRF-Ala-AGC in M2 macrophages can activate the NF-κB signaling pathway in BC cells 9. Furthermore, the association of tsRNA with exosomes has been implicated in tumor immunity, with tRF-GluCTC-0005 in pancreatic cancer-derived exosomes being shown to be important for recruiting myeloid-derived suppressor cells to a tumor site, leading to the formation of an immunosuppressive microenvironment 108. The molecular mechanism for the actions of tRF-GluCTC-0005 in this cellular context has been found to involve its binding to the 3' untranslated region of WDR1 mRNA in hepatic stellate cells, leading to stabilization of this mRNA, as well as the activity of YAP, a modulator of cellular gene expression 108.

### The roles for tsRNA in autoimmune disorders

Systemic lupus erythematosus (SLE) is an autoimmune disorder that is associated with a loss of nuclear and cytoplasmic self-antigens, leading to the production of autoantibodies and the formation of immune complexes 109, 110. These factors contribute to inflammation that affects multiple organ systems. Recent research has found that tsRNAs may serve as diagnostic biomarkers for SLE that are detected in the sera, particularly when changes to levels of tRF-His-GTG-1 and levels of anti-dsDNA are observed 111. Moreover, serum tsRNAs have been shown to directly target signaling molecules that play a pivotal role in the regulation of the immune system 111. Furthermore, tsRNAs are differentially expressed in peripheral blood mononuclear cells from SLE patients compared to healthy controls. Bioinformatic analysis of these differences suggests that the altered target genes are prominently enriched in T cell receptor signaling pathways, Th1 and Th2 cell differentiation, and primary immunodeficiency 112. Another study of tsRNAs in SLE found that, mesenchymal stem cell exosomal tsRNA-21109 could ameliorate SLE by inhibiting macrophage M1 polarization 11, 113. In addition, research by Geng *et al.* demonstrated that tsRNAs, such as tRF-3009, were involved in the modulation of IFN-α-induced oxidative phosphorylation in CD4+ T cells of lupus patients. *In vitro* analysis of CD4+ T cells overexpressing tRF-3009 revealed a correlation between this product and type I IFN (interferon) as well as oxidative phosphorylation pathways. Notably, IFN-α demonstrated the capacity to stimulate the generation of reactive oxygen species (ROS) and ATP in CD4+ T cells. Conversely, the knockdown of tRF-3009 reversed these effects. Overexpression of tRF-3009 alone in CD4+ T cells was sufficient to enhance oxygen consumption rate, ROS production, and ATP generation 114.

Sarcoidosis is a systemic autoimmune disease characterized by the formation of non-caseating granulomas, a pathological hallmark of the condition as noted in recent studies 115. Notably, three specific tsRNAs, namely tiRNA-Glu-TTC-001, tiRNA-Lys-CTT-003, and tRF-Ser-TGA-007, were reported to be significantly dysregulated in sarcoidosis, and suggest that these tsRNAs mediate the pathophysiology of this disease 116. A further bioinformatics study revealed the possible involvement of such tsRNAs in mediating chemokine signaling, cAMP- and cGMP-PKG pathways, retrograde endorphin signaling, and the FoxO pathway 116. Despite these advances in knowledge, the precise mechanisms by which these tsRNAs modulate the immune response in sarcoidosis remain to be defined.

### The involvement of tsRNAs in cellular infections

Recent studies has illuminated the involvement of tsRNAs in viral infections 59, 117, as evidenced by the promotion of RSV replication by 5'-tRF-Gly-CCC and 5'-tRF-Lys-CTT 118. In patients diagnosed with COVID-19, caused an infection by the severe acute respiratory syndrome coronavirus 2 (SARS-CoV-2), tsRNAs, particularly from the tRF-5, have been detected in nasopharyngeal swabs of affected individuals 119. Intriguingly, post-SARS-CoV-2 infection, an upregulation of specific tsRNAs has been observed in the blood, with higher levels correlating with the severity of COVID-19 symptoms. Specifically, the 3'CCA tsRNAs derived from tRNA-Gly have been found to be significantly associated with the inflammatory marker C-reactive protein, and could therefore present as a possible target for therapeutic modulation 120. In bovine studies, dysregulation of five tsRNAs (tRF-36-8JZ8RN58X2NF79E, tRF-20-0PF05B2I, tRF-27-W4R951KHZKK, tRF-22-S3M8309NF, and tRF-26-M87SFR2W9J0) in calves infected with bovine leukemia virus is indicative of their association with such an infection 121. In addition, tsRNAs have been implicated in bacterial infections. For example, Gumas *et al.* reported a global and substantial upregulation of plasma small non-coding RNAs (sncRNAs) in patients infected with mycobacterium tuberculosis (MTB), with tsRNAs being the most significantly elevated class 122. These sncRNAs, which are notably abundant in MTB-infected patients, potently activate human macrophages via TLR7, triggering cytokine production 122. These findings collectively offer insight into how tsRNAs might influence the immune response when challenged by an infectious disease, and reveals putative tsRNA candidates that could be modulated for therapeutic impact.

### The roles for tsRNAs in other health conditions

The modulation of the immune system by tsRNAs has been observed in a spectrum of health challenges. For example, in hypertrophic scarring, the overexpression of tsRNA-14783 is linked to macrophage polarization towards the M2 phenotype, and this, in turn, has been reported to influence scar formation through the upregulation of M2 macrophage markers such as TGF-β, IL-10, and CD206, and downregulation of M1 macrophage markers like IL-1 and NOS2 123, 124. In the context of diabetes, it has been reported that alterations to tsRNAs profiles within the islets of NOD mice are observed during the early stages of type 1 diabetes. A subset of these tsRNAs, including Gly-GCC-5'H and Leu-CCA-I that are enriched in EVs from CD4+/CD25- T cells, impacts beta cell gene expression and immune regulation, and predisposing them to apoptosis, and this suggests a role for tsRNAs in diabetes pathogenesis 125. In another scenario, high-throughput sequencing studies have identified differential tsRNA expression (tDR-006826, tDR-006049, tDR-001271 and tDR-001276) in bone marrow mesenchymal stem cells of patients with fibrous dysplasia (FD), a disease characterized by abnormal bone tissue replacement. These tsRNAs were associated with immune response regulation, as revealed by GO and KEGG pathway analysis 126. In renal ischemia-reperfusion injury, differential tsRNA expression, including tiRNA-Gly-GC-003, tiRNA-Lys-CTT-003, and tiRNA-His-GTG-002, has been associated with natural killer cell-mediated cytotoxicity pathways, and this could suggest that tsRNAs could influence acute kidney injury progression following ischemia-reperfusion 127. In pigs, intrauterine growth restriction is marked by differential tsRNA expression, with tRF-5c predominating and originating mostly from tRNA-Gly-GCC. Other significant tsRNAs, including tiRNA-Ser-TGA-001 and tRF-Val-AAC-034, have also been implicated in immune system processes, with predicted target genes including TNF, TLR4, CD44, MAPK1, and STAT1, potentially regulating T cell receptor and toll-like receptor signaling pathways 128. In the context of post-muscle injury, 5'tiRNA-Gly is upregulated and correlates with inflammation, promoting pro-inflammatory cytokines (IL-1β, IL-6) and M1 macrophage markers (TNF-α, CD80, MCP-1), increasing the percentage of CD86+ macrophages and inhibiting the percentage of CD206+ macrophages. Notably the mechanism of action for 5'tiRNA-Gly appears to involve modulation of the gene Tgfbr1 in an AGO1- and AGO3-dependent manner. Through modulation of the TGF-β signaling pathway, 5'tiRNA-Gly regulates the expression of downstream genes related to inflammation, satellite cell activation, and myoblast differentiation. These findings indicate that 5'tiRNA-Gly may modulate skeletal muscle regeneration through TGF-β-mediated inflammation, and this, in turn, suggests that 5'tiRNA-Gly may be a viable target for the treatment of skeletal muscle regeneration 129.

**Figure 4:**
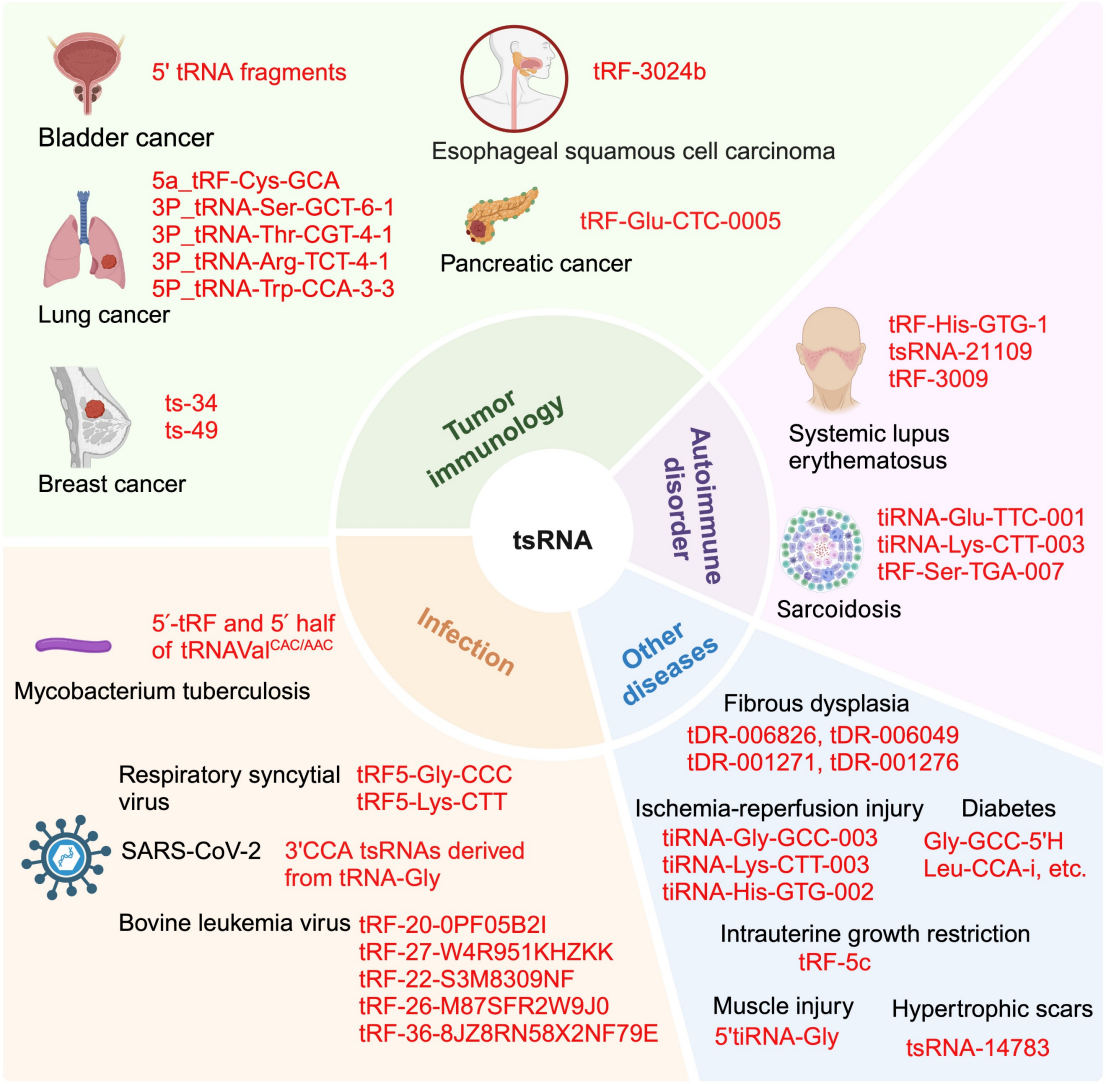
** tsRNA's involvement in diverse immune-related diseases.** tsRNA plays a role in various immune-related conditions, including different types of cancer such as bladder cancer, lung cancer, braest cancer, esophageal squamous cell carcinoma and pancreatic cancer. It is also implicated in autoimmune diseases like systemic lupus erythematosus and and sarcoidosis. Additionally, tsRNA is associated with bacterial and viral infections, including respiratory syncytial, SARS-Cov-2 and bovine leukemia virus. Other conditions where tsRNA is involved encompass hypertrophic scars, intrauterin growth restriction, diabetes, ischemia-reperfusion injury, muscle injury and fibrous dysplasia. This figure was created with BioRender.com.

A comprehensive overview of the functions of tsRNAs in across multiple diseases and in the context of immunity is presented in Table 1, and a schematic representation of the role for tsRNAs in disease immunity is provided in Figure 4.

## Conclusion and future perspectives

Here, we have reviewed the evidence for tsRNAs as pivotal regulators in a spectrum of biological processes, including in immune responses and in the pathogenesis of a wide range of diseases. The intricate biogenesis of tsRNAs and their diverse functionalities, particularly in immunological contexts, are increasingly being recognized. Despite these advancements, there is a sense that we are only beginning to understand how tsRNAs carry out its cellular functions to health and disease. Notably, the accurate detection of tsRNAs remains a significant challenge due to their small size, while the presence of modifications on tsRNAs can interfere with RNA-seq approaches for detecting these non-coding RNAs. However, with the recognition of the pivotal roles that small RNAs play in both physiological and pathological processes, there has recently been a surge in the development of improved sequencing technologies that have been specifically designed to detect small RNAs with high sensitivity and specificity. Such approaches include CPA-seq 130, PANDORA-seq 16 and AQRNA-seq 131, all of which hold promise to improve detection of tsRNAs. Additionally, post-sequencing, it is imperative to verify whether these tsRNAs are mere degradation products or possess functional roles.

The clinical application of tsRNAs in immunity shows promise, with evidence that tsRNAs could be targeted as biomarkers or as therapeutic targets. For example, tsRNAs identified in conditions such as cancer and autoimmune disorders could be targets for restoring immune balance. In cancer, where immune evasion is a critical hallmark, tsRNAs could be leveraged to enhance the efficacy of immunotherapies, whereby immunosuppressive tsRNAs could be inhibited, while the activity of tsRNAs that promote anti-tumor immunity could be boosted. In the context of autoimmune diseases, tsRNAs could be used to suppress an overactive immune response.

Moreover, when considering infectious diseases, tsRNAs could be leveraged to enhance the immune defense. We surmise that potential clinical applications of tsRNAs in the context of disease immunity can be summarized as follows: firstly, as immune-modulators, certain 5'-tRNA halves from tRNA-His-GUG that activate TLR7, could be key as immune activators 92, 93. Secondly, the levels of tsRNAs could be predictive of the immune status of patients and possible be prognostic for disease severity, such as for BC, where T-cell activation is significantly associated with ts-34 and ts-49 104. Thirdly, tsRNAs could be investigated as therapeutic targets. For example, knockdown of tRF-3009 in lupus patients' CD4+ T cells reverses IFN-α-induced oxidative phosphorylation and this approach could be effective as a strategy to develop this tsRNA as therapeutic targets for SLE 114. Fourthly, tsRNA agonists may be developed as an exosome-delivered therapeutic, since it has been shown that exosomal tsRNA-21109 from mesenchymal stem cells can SLE by inhibiting macrophage M1 polarization 11. Finally, the design of inhibitors that target tsRNAs could enhance the efficacy of immunotherapies. For example, in ESCC, tRF-3024b enhances the tolerance of ESCC to CTLs, suggesting that inhibitors could be designed to increase tumor cell sensitivity to CTLs 107.

In conclusion, we find that tsRNAs are relevant to disease immunity, and there remains a clear need for clarification of the mechanistic functions of tsRNAs in health and disease, particularly in the context of immune system interactions. Future research is also needed to corroborate bioinformatics predictions and explore the translational potential of tsRNAs within clinical settings. Moreover, the stability of tsRNAs within the immune microenvironment and their potential for targeted delivery as therapeutic agents are also important areas for research. As our understanding of tsRNA biology expands, so too does the opportunity to harness their potential in managing tissue homeostasis, as well as immune-related diseases and other health challenges.

## Figures and Tables

**Table 1:** The role of tsRNAs in disease immunity

Disease	tsRNA	Function	Mechanism	Reference
Breast cancer	ts-34, ts-49	Relates to T cell activation status.	\	104
	3'tRF-Ala-AGC	Promotes cell malignant activity and facilitates M2 polarization of macrophages.	Binds Type 1-associated death domain protein, and its overexpression in M2 macrophages activates NF-κB signaling pathway in BC cells.	9
Lung cancer	5a_tRF-Cys-GCA, 3P_tRNA-Ser-GCT-6-1, 3P_tRNA-Thr-CGT-4-1, 3P_tRNA-Arg-TCT-4-1, 5P_tRNA-Trp-CCA-3-3	Positively associates with PD-L1 immune checkpoint and correlates with genes that target in PD-L1 checkpoint signaling pathway.	\	105
Prostate cancer	5' tsRNAs due to METTL1 depletion	Suppresses prostate tumour growth, and enhances the response to immune checkpoint blockade therapy.	Represses translation initiation, and activates IFN signalling pathway.	106
Esophageal squamous cell carcinoma	tRF-3024b	Reduces the apoptosis of tumor cells when co-cultured with cytotoxic T lymphocytes.	Promotes the expression of B-cell lymphoma-2 by sequestering miR-192-5p, a microRNA that would normally inhibit BCL-2 expression.	107
Pancreatic cancer	tRF-GluCTC-0005	tRF-GluCTC-0005 in pancreatic cancer-derived exosomes recruits myeloid-derived suppressor cells in liver, and creates an immunosuppressive microenvironment, as well as advances liver metastasis from pancreatic cancer.	Binds to the 3' untranslated region of WDR1 mRNA in hepatic stellate cells, stabilizing the mRNA and affecting the YAP protein's activity.	108
Systemic lupus erythematosus	Differentially expressed tsRNAs	Enriched in T cell receptor signaling pathways, Th1 and Th2 cell differentiation, and primary immunodeficiency.	\	112
	tsRNA-21109	tsRNA-21109-privative MSC-exo upregulates M1 markers, downregulates M2 markers, and increases levels of TNF-α and IL-1β in macrophages.	\	11
	tRF-3009	Positively correlates with SLE disease activity index, active lupus nephritis and serum IFN-α levels.	Upregulates IFN-α-induced ROS and ATP production in CD4+ T cells.	114
Sarcoidosis	tiRNA-Glu-TTC-001, tiRNA-Lys-CTT-003, tRF-Ser-TGA-007	May play roles in chemokine, cAMP, cGMP-PKG, retrograde endorphin, and FoxO signalling pathways by bioinformatics analyses.	\	116
post-SARS-CoV-2 infection	3'CCA tsRNAs derived from tRNA-Gly	Associates with the inflammatory marker C-reactive protein.	\	120
MTB infection	5'tsRNAs derived from tRNA-His-GUG, tRNA-Glu-CUC, tRNA-Val-CAC/AAC	upregulated in patients infected with MTB, and could be as potent activators of macrophage TLR7.	\	122
Hypertrophic scarring	tsRNA-14783	Promotes scar formation by regulating macrophage polarization towards the M2 phenotype.	\	123, 124
Diabetes	Gly-GCC-5'H, Leu-CCA-i, etc.	Impacts beta cell gene expression and immune regulation, predisposing them to apoptosis	These tsRNAs in EVs from CD4+/CD25- T cells move into beta cells through adoptive transfer.	125
Fibrous dysplasia	tDR-006826, tDR-006049, tDR-001271, tDR-001276	Involved in immune response regulation, as revealed using bioinformatics analyses.	\	126
Renal ischemia-reperfusion injury	tiRNA-Gly-GC-003, tiRNA-Lys-CTT-003, tiRNA-His-GTG-002	Associates with natural killer cell-mediated cytotoxicity pathways, as reveaed using bioinformatics analyses.	\	127
Intrauterine growth restriction	tiRNA-Ser-TGA-001, tRF-Val-AAC-034	Mediates the immunocompromise caused by intrauterine growth restriction.	May target TNF, TLR4, CD44, MAPK1, and STAT1, potentially regulate T cell receptor and toll-like receptor signaling pathways	128
Muscle injury	5'tiRNA-Gly	Upregulated after muscle injury and positively correlated with inflammation; promotes expression of proinflammatory cytokines, the M1 markers of macrophages, the percentages of CD86+macrophages, and myogenic differentiation marker genes of myoblast.	Targets TGFBR1 in a AGO1- and AGO3-dependet manner to regulate the TGF-β signaling pathway.	129

“\” indicates that the molecular mechanism is currently unknown
